# Non-enhancing bowel dilatation and secondary ileus on CT as a surgical red flag in geriatric patients with atypical presentations

**DOI:** 10.1186/s12877-025-06431-5

**Published:** 2025-09-29

**Authors:** Xirang Wang, Jian Kang, Yuxiang Li, Xiaofeng Sun, Yunpeng Wu, Jun Zhang, Hehui Tao, Li Wang, Ruizhou Rong, Miao Wang, Kang Liu, Zhen Ban

**Affiliations:** 1 Department of General Surgery, Beijing Fengtai Youanmen Hospital, Beijing , 100069 China; 2 Department of General Surgery, Beijing Huimin Hospital, Beijing , 100054 China

**Keywords:** Ischemic bowel disease, Pneumatosis coli, Intestinal necrosis

## Abstract

**Background:**

Ischemic bowel disease represents a critical diagnostic challenge in emergency surgical practice. Despite its relatively low incidence in patients presenting with abdominal pain, this condition demands urgent attention due to its potentially fatal outcomes when management is delayed.

**Case presentation:**

We present a clinically instructive case of a nonagenarian female initially diagnosed with colonic pneumatosis through imaging studies. Subsequent diagnostic reevaluation revealed progressive colonic necrosis requiring emergent surgical intervention. The patient underwent successful segmental colectomy with colostomy, achieving full recovery and discharge within 14 postoperative days.

**Conclusion:**

This case highlights three critical aspects in managing geriatric patients with acute abdominal emergencies: The inherent diagnostic limitations posed by atypical presentations in elderly populations, including unreliable history-taking and attenuated physical signs; The insufficient sensitivity of conventional laboratory markers (leukocytosis, NEUT%, PCT, lactate elevation) for detecting intestinal ischemia; The pivotal role of contrast-enhanced computed tomography (CT) in surgical decision-making, particularly the prognostic significance of non-enhancing bowel wall dilatation as a radiographic hallmark of transmural necrosis. Early surgical consultation and protocolized CT interpretation are paramount for optimizing outcomes in this high-risk patient cohort.

## Background

Colonic pneumatosis intestinalis (CPI), a rare clinical entity pathologically defined by gas-filled cystic structures within the submucosal or subserosal layers of the bowel wall [1, 2], is generally managed conservatively in hemodynamically stable patients without peritoneal signs or significant laboratory derangements [3]. In contrast, mesenteric ischemia—though accounting For only 0.09–0.2% of acute abdominal emergencies requiring surgical admission [4]—carries grave prognostic implications. Epidemiologically, colonic ischemia predominates among mesenteric vascular pathologies, with computed tomography (CT) angiography and endoscopic biopsy constituting the diagnostic mainstays [5]. Anatomical vulnerability arises from three zones with minimal collateral circulation (splenic flexure, rectosigmoid junction, and ileocecal region), which are predisposed to non-occlusive ischemic injury during systemic hypoperfusion [6]. Mesenteric ischemia has a median age of onset of approximately 70 years, with a mortality rate ranging from 50 to 66% [7]. Even in some studies [8], the mortality rate is as high as 80%. Despite the advancements in radiology, vascular surgery and critical care medicine, the mortality rate and intestinal resection rate have remained unchanged for decades [9].

This case exemplifies the treacherous diagnostic landscape of ischemic bowel disease in elderly patients, where confounding factors such as blunted peritoneal signs, nonspecific biomarkers, and comorbidities often delay life-saving interventions. Through multidisciplinary synthesis of imaging findings, surgical exploration, and evidence-based protocols, we demonstrate critical strategies to mitigate mortality in this high-risk demographic.

## Main text and case presentation

A 90-year-old female with multiple comorbidities (30-year history of type 2 diabetes with peripheral neuropathy, coronary artery disease, hypertension, dyslipidemia, prior lumbar spine surgery, and left femoral head arthroplasty) presented to our emergency department on November 18, 2024, with a 22-hour history of intermittent colicky abdominal pain localized to the epigastrium and periumbilical region. Associated symptoms included 4–5 episodes of non-bilious vomiting and 3 loose non-bloody bowel movements.

**Fig. 1:**
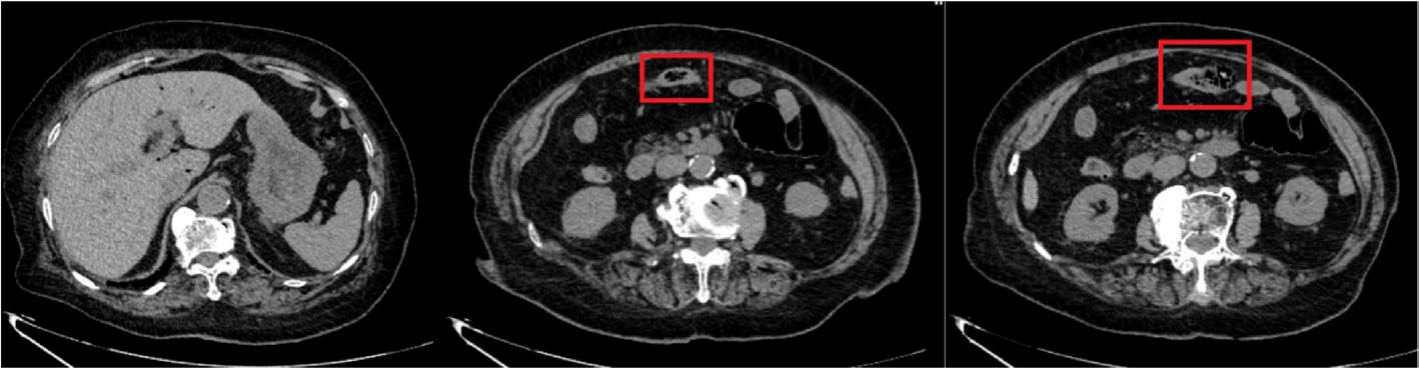
Initial Non-Contrast Abdominal CT (External Institution, November 17, 2024)

**Fig. 2:**
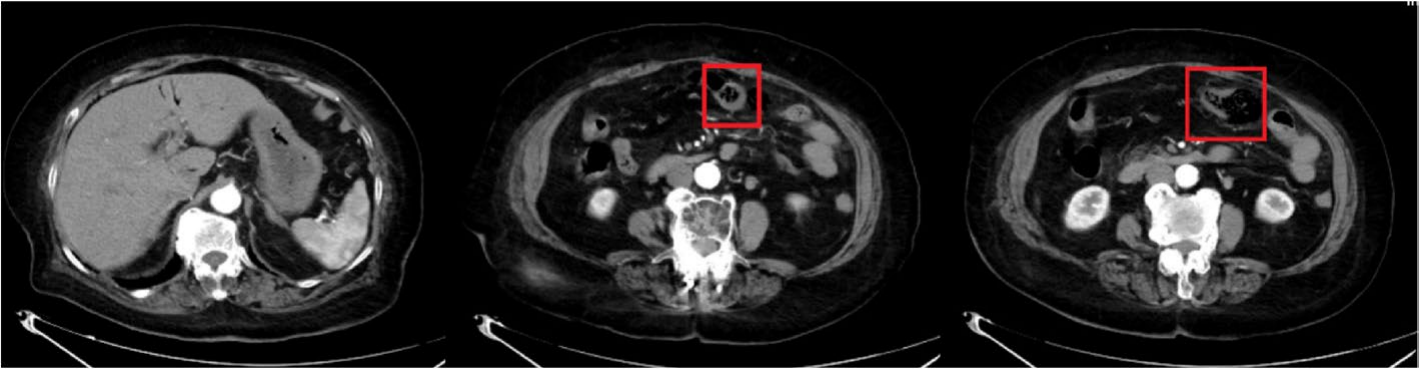
Contrast-enhanced Abdominal CT (External Institution, November 17, 2024)

Initial laboratory studies from a referring hospital (November 17) revealed leukocytosis (12.26 × 10⁹/L; neutrophil predominance 93.9%) with normal C-reactive protein (< 0.5 mg/L). Non-contrast abdominal CT demonstrated pneumatosis intestinalis at the splenic flexure, gas infiltration within the mesentery adjacent to the transverse colon, and portal venous gas - findings initially attributed to ruptured colonic pneumatosis (Fig. 1). Subsequent contrast-enhanced CT showed persistent intramural gas with partial resolution of portal venous gas, raising suspicion for ischemic colitis (Fig. 2).

Upon admission to gastroenterology, serial monitoring revealed progressive systemic inflammation (November 18: WBC 14.22 × 10⁹/L, CRP 43.46 mg/L, procalcitonin 0.56 ng/mL, IL-6 976.07 pg/mL) despite stable abdominal findings (deep epigastric tenderness without rebound, normoactive bowel sounds). Conservative management with bowel rest, broad-spectrum antibiotics (Cefoperazone-Sulbactam Sodium), and parenteral nutrition was initiated.

**Fig. 3:**
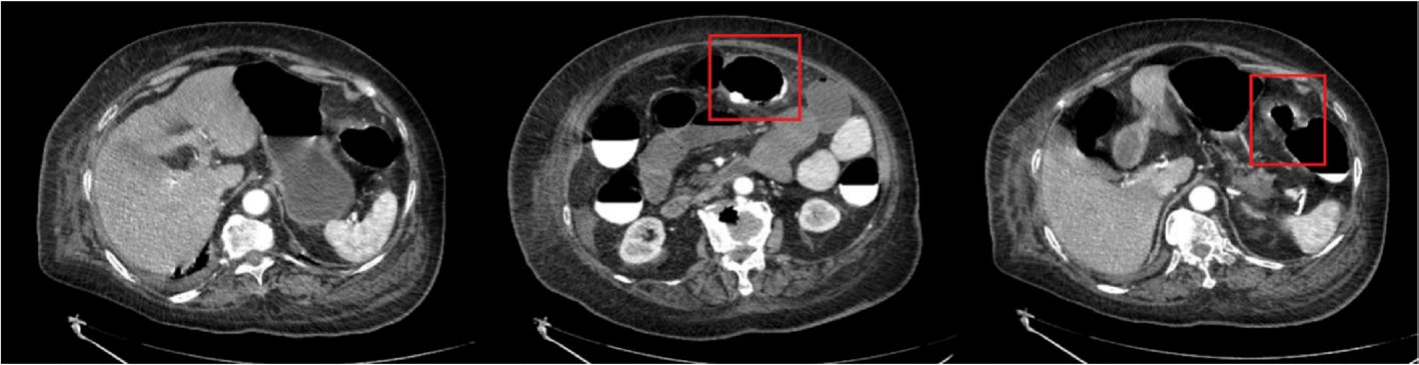
Contrast-Enhanced Abdominal CT (November 20, 2024)

By November 20, the patient developed worsening abdominal pain accompanied by dramatic CRP escalation to 162.42 mg/L. Repeat CT angiography revealed: Non-enhancing mural thickening of the transverse colon-splenic flexure continuum, progressive pneumatosis with mesenteric stranding, small bowel and colonic dilatation with air-fluid levels, patent superior mesenteric artery and attenuated inferior mesenteric artery (Fig. 3).

**Fig. 4:**
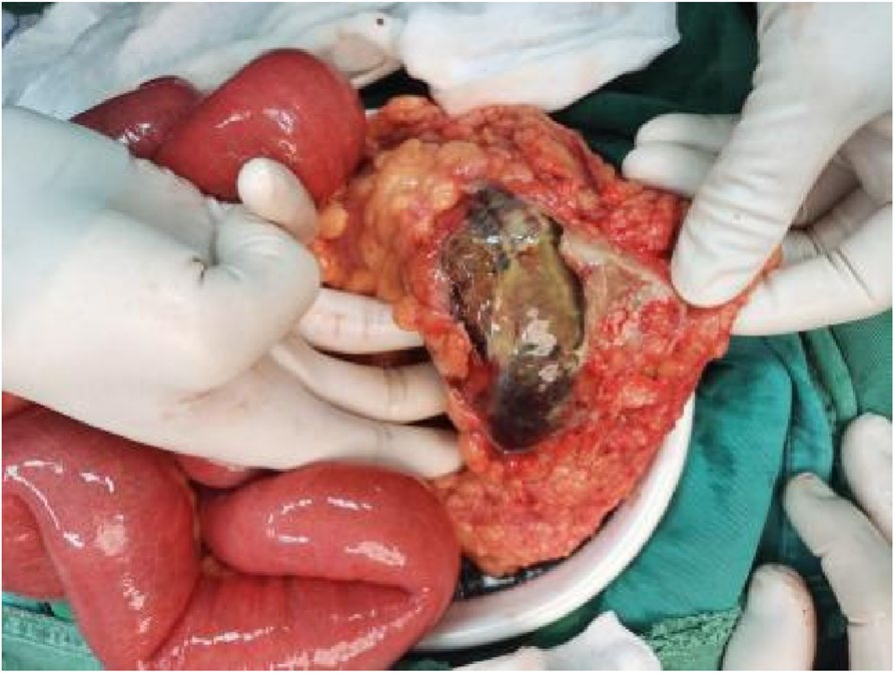
Intraoperative exploration showed partial necrosis of the transverse colon (November 20, 2024)

General surgery consultation was invited, Emergency general surgery consultation identified three critical red flags: Inflammatory indicators increase progressively; CT evidence of non-perfused bowel (absence of mural enhancement); Failure of conservative therapy despite 48-hour intensive monitoring. After general surgery consultation and multidisciplinary evaluation, surgical intervention was determined to be necessary. The patient was transferred to the general surgery ward and underwent exploratory laparotomy on November 20, 2024. Upon initial entry into the abdominal cavity, no inflammatory reaction, effusion, or pus was observed. However, upon retracting the omentum superiorly, segmental necrosis of the splenic flexure of the transverse colon was identified, along with surrounding exudate, as well as dilated and swollen ileum and ascending colon (Fig. 4). Subsequently, a necrotic colon resection with proximal colostomy was performed. After the operation, the patient was transferred to the ICU due to ventilator dependence. After anti-infection, nutritional support and cardiopulmonary function regulation, the patient recovered and was discharged on the 14th day after the operation.

### Discussion and conclusions

The patient, presenting with intestinal obstruction and significant abdominal distension, underwent open exploratory laparotomy without prior laparoscopic assessment due to restricted abdominal space. Initial intraoperative exploration revealed a clean abdomen without inflammation, explaining the absence of preoperative peritonitis signs. Further examination showed localized necrotizing colitis in the transverse colon, encapsulated by the omentum and mesentery, restricting infection spread and accounting for the patient’s atypical presentation of no fever and slow - progressing inflammatory markers.

The transverse colon’s blood supply mainly comes from the middle colic artery, a superior mesenteric artery branch, with additional supply from the marginal artery, an anastomotic arch between the left and right colic arteries. The colon has a relatively low blood flow and reduced perfusion during functional inactivity, making it vulnerable to ischemia [10]. The splenic flexure has few vascular collaterals, being the most susceptible to ischemic changes when blood flow decreases. The colonic mucosa and submucosa receive 70% of the mesenteric blood, while the muscularis propria and serosa receive the remaining 30%. Ischemic injury first occurs in the colonic mucosa, most commonly on the anti - mesenteric side [5].

The patient had multiple ischemic colitis risk factors: type 2 diabetes with peripheral neuropathy, hypertension, and hyperlipidemia [11], along with age - related vascular sclerosis [12]. Combined with the splenic flexure’s blood supply characteristics, this made the area prone to ischemic necrosis. The early appearance of gas shadows in the colon’s submucosa, serosa, adjacent mesentery, intra - and extrahepatic bile ducts, and portal vein suggested early - stage intestinal ischemia with mucosal necrosis and gas leakage, rather than colonic pneumatosis cystoides. Currently, there are no reported cases of colonic pneumatosis cystoides - related bowel necrosis.

Ischemic colitis, though rare, carries high mortality. Its diagnosis is challenging due to the absence of specific laboratory markers and the frequent occurrence of atypical presentations in geriatric patients—attributable to physiological decline, comorbidities, and blunted peritoneal signs that delay clinical manifestations [13]. It often presents with elevated white blood cell counts, hyperlactatemia, and positive D - dimer [5]. The clinical severity of ischemic injury is hard to determine due to infrequently reported grading in studies [14]. Among CT features, the absence of bowel wall enhancement is a better diagnostic indicator for transmural necrosis [15]. Bowel dilation, recently identified as the most consistent CT feature of transmural necrosis [16], was present in this case. Especially when inflammatory markers are inconclusive, non - enhanced CT throughout the bowel wall and bowel obstruction can serve as specific indicators for bowel necrosis diagnosis. The presence of CT features diagnostic of bowel necrosis—specifically non-enhancing mural dilatation and obstruction—should mandate surgical exploration irrespective of peritoneal signs, particularly in frail geriatric patients [17, 18]. Delayed intervention in this high-risk cohort escalates mortality due to rapid progression to perforation or sepsis. Thus, CT evidence of transmural necrosis constitutes an absolute indication for surgery, overriding the absence of classical physical findings. In cases with severe bowel dilatation and abdominal distension, laparoscopic exploration is often precluded. Here, open laparotomy—including planned second-look laparotomy—becomes imperative to resect necrotic segments promptly, preventing perforation or death [19]. Current evidence supports intervention within 48–72 h to avert disease progression and mitigate poor outcomes [13]. Indocyanine green (ICG) fluorescence imaging serves as a safe, real-time adjunct for intraoperative bowel perfusion assessment [20]. In acute abdominal emergencies (e.g., bowel obstruction, mesenteric ischemia, or incarcerated hernias), ICG accurately discriminates salvageable ischemic segments from nonviable tissue, thereby avoiding unnecessary resections or missed necrosis [21, 22]. Intraoperatively, the affected bowel segment exhibited complete loss of normal tissue architecture with unequivocal transmural necrosis, as evidenced in Fig. 4. Consequently, ICG fluorescence imaging was not employed since perfusion assessment becomes irrelevant in the presence of definitive non-viability.

Retrospectively analyzing this elderly patient’s transverse colon necrosis case, the CT features of non - enhanced bowel wall and obstruction are valuable for timely and accurate diagnosis in atypical cases with inconclusive inflammatory markers. This enables early surgical intervention, preventing disease progression and reducing mortality risk.

## Data Availability

The datasets used and/or analyzed during the current study are available from the corresponding author on reasonable request.

## Abbreviations

CT: Computed Tomography
NEUT: Neutrophil
PCT: Procalcitonin
CPI: Colonic Pneumatosis Intestinalis
ICU: Intensive Care Unit
D-dimer: Fibrin degradation product D-dimer
ICG: Indocyanine Green
